# Kartagener Syndrome in Two Siblings: A Familial Case Report With Phenotypic Variability and Destroyed Lung Syndrome as a Rare Complication

**DOI:** 10.7759/cureus.88375

**Published:** 2025-07-20

**Authors:** Imane El Khachine, Abdelkader Boukharta, Rachida Zahraoui, Jamal Eddine El Bourkadi, Mouna Soualhi

**Affiliations:** 1 Pulmonology Department, Moulay Youssef Hospital, Centre Hospitalo-Universitaire (CHU) Ibn Sina, Rabat, MAR; 2 Respiratory Department, Hospital Mohammed VI University Hospital Center, Tangier, MAR

**Keywords:** bronchiectasis, destroyed lung syndrome, familial case, kartagener syndrome (ks), primary ciliary dyskinesia (pcd), rare complication, situs inversus totalis (sit)

## Abstract

Kartagener syndrome is a rare genetic disorder characterized by the classic triad of situs inversus, bronchiectasis, and chronic sinusitis. The progression to destroyed lung syndrome represents an exceptionally rare complication, with only a few cases reported in the literature.

We present the cases of two Moroccan sisters, aged 20 and 26, born from a first-degree consanguineous marriage, presenting with chronic respiratory symptoms but with different phenotypes. The first patient had a severe phenotype including destroyed lung syndrome, chronic bronchitis, recurrent infections, and imaging findings of dextrocardia with extensive lung tissue destruction. The second patient presented with chronic cough, anosmia, and bronchiectasis with less severe pulmonary involvement than her sibling. Imaging studies confirmed complete situs inversus in both cases. Diagnosis relies on clinical and radiological criteria.

These cases highlight the phenotypic variability of Kartagener syndrome in the same family and the possibility of severe pulmonary involvement leading to a completely destroyed lung, a very rare but possible complication. Hence, it is important to regularly screen patients for lung parenchymal destruction and regularly monitor those with severe radiographic changes.

## Introduction

Kartagener syndrome (KS) is defined by the triad of bronchiectasis, chronic sinusitis and situs inversus. It is a rare autosomal recessive disorder classified under primary ciliary dyskinesia (PCD) [1], with an estimated incidence of approximately one case per 32,000 live births. PCD represents a clinically and genetically heterogeneous group of respiratory ciliopathies, characterized by impaired mucociliary clearance, leading to recurrent lower respiratory infections [2]. Clinical presentation and severity may vary from one individual to another, even within the same family [2,3]. However, progression to a destroyed lung, which is an extensive and irreversible pulmonary parenchymal destruction, is extremely rare in KS.

Destroyed lung syndrome is a clinico-radiological condition characterized by complete destruction and severe volume loss of one lung, often resulting from chronic infections such as tuberculosis or severe bronchiectasis. Pulmonary function may be initially preserved due to compensatory hyperinflation of the contralateral lung; however, chronic respiratory failure can progressively develop over time [4].

We report the cases of two sisters born from a consanguineous marriage, presenting different phenotypic expressions of Kartagener syndrome, with the younger sister complicated by a completely destroyed lung.

## Case presentation

Case 1

S.S., a 20-year-old Moroccan female, was born of a first-degree consanguineous marriage. She had no history of tuberculosis (TB) or recent TB contact. The patient had no history of cigarette smoking, vaping, or any specific environmental exposures such as contact with birds, pets, or organic dusts. She was hospitalized at day 15 of life in the neonatal unit for respiratory distress. She had a history of chronic bronchitis with recurrent respiratory infections since childhood.

For the past month, she had been experiencing a productive cough with purulent sputum, associated with dyspnea on exertion. She was initially treated in an outpatient setting with empirical antibiotic therapy (amoxicillin-clavulanic acid for seven days), without improvement, leading to her admission to the Department of Pulmonology for further care.

On clinical examination, the patient was eupneic with a respiratory rate of 18 cycles per minute. Oxygen saturation (SpO2) was 95%. She had fever at 37.7°C. No digital clubbing was noted. Pulmonary auscultation revealed diffuse rhonchi over the right hemithorax. Heart sounds were heard on the right.

The otolaryngological examination and anterior rhinoscopy revealed hypertrophied inferior turbinates with mucosal congestion and polyps consistent with chronic rhinosinusitis with nasal polyposis.

**Figure 1:**
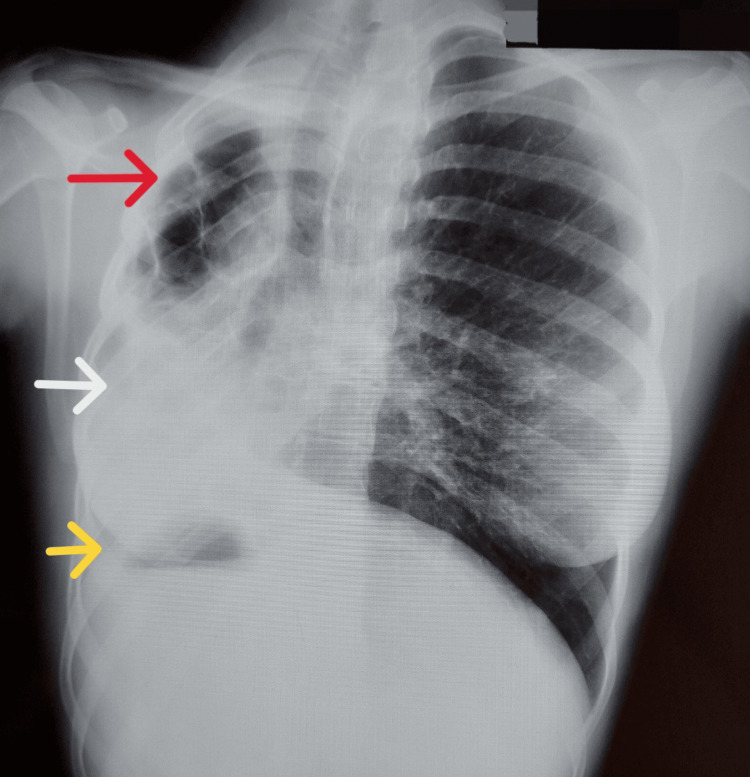
Chest X-ray The chest X-ray showed significant lung damage (red arrow) with rightward deviation of the trachea and cardiac silhouette (white arrow), with left lung hyperinflation. The stomach bubble is on the right side (yellow arrow), suggesting situs inversus.

Chest X-ray showed significant lung damage with rightward deviation of the trachea and cardiac silhouette, with left lung hyperinflation. The stomach bubble was on the right side, suggesting situs inversus (Figure 1).

**Figure 2:**
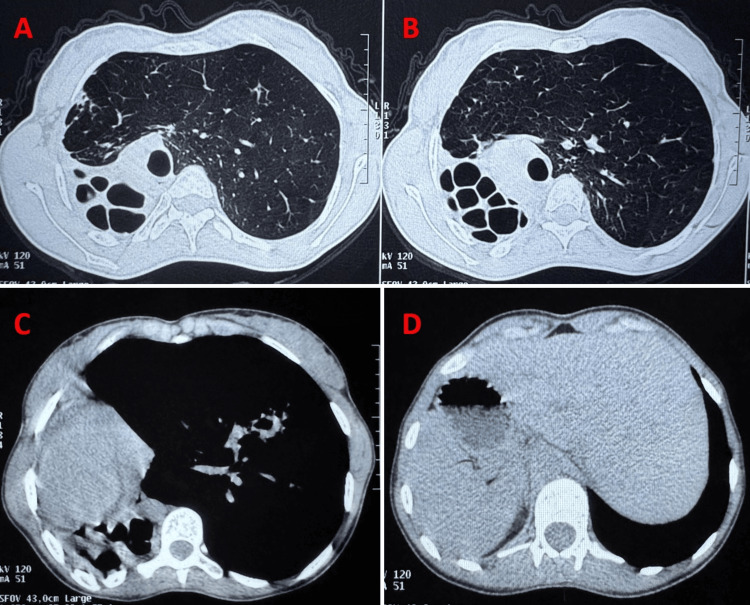
Chest CT scan Thoracic CT scan confirmed complete situs inversus (dextrocardia, right-sided spleen, and left-sided liver)(C and D) with cystic bronchiectasis occupying the right lung, associated with bullous dystrophy and significant parenchymal destruction (A and B).

**Figure 3:**
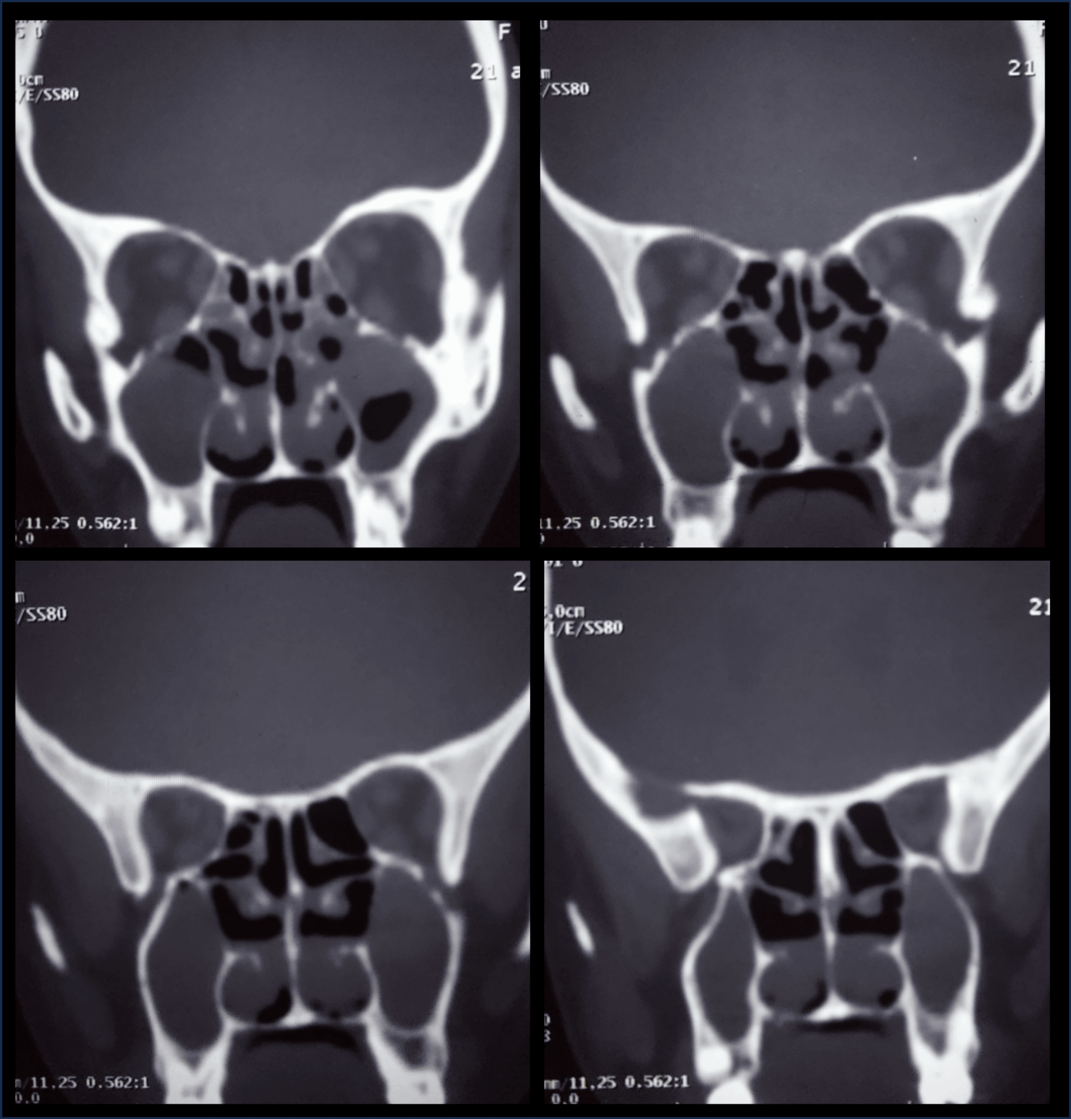
Sinus CT scan showed acute pansinusitis on a background of chronic sinus disease.

Thoracic CT confirmed complete situs inversus (dextrocardia, right-sided spleen, and left-sided liver) with cystic bronchiectasis occupying the right lung, associated with bullous dystrophy and significant parenchymal destruction. The left lung showed cylindrical bronchiectasis with signs of mucus impaction (Figure 2). Sinus CT scan showed acute pansinusitis on a background of chronic sinus disease (Figure 3).

Biological tests revealed an inflammatory syndrome, with a white blood cell count of 13,400/mm³ and a C-reactive protein level of 70 mg/L. Acid-fast bacilli (AFB) smear and GeneXpert (Cepheid, Sunnyvale, CA, USA) testing for tuberculosis were negative. Aspergillus serology was negative. Sputum culture identified Pseudomonas aeruginosa as the causative pathogen (Table 1).

**Table 1:** Biological test results AST: aspartate aminotransferase; ALT: alanine aminotransferase; MTB: Mycobacterium tuberculosis; RIF: rifampicin

Test	Result	Units
White blood cells (WBCs)	13.4	×10³/µL
Polymorphonuclear Neutrophils (PMN)	10.0	×10³/µL
Hemoglobin (HGB)	11.9	g/dL
Hematocrit	36.1	%
Platelets (PLT)	367	×10³/µL
C-reactive protein (CRP)	70	mg/L
Urea	0.29	g/L
Serum creatinine	7.00	mg/L
AST (SGOT)	11.0	U/L
ALT (SGPT)	8.00	U/L
Gamma-GT	12	U/L
Total bilirubin	4.00	mg/L
Indirect bilirubin	2.00	mg/L
Direct bilirubin	2.00	mg/L
Sodium (Na⁺)	138	mmol/L
Potassium (K⁺)	3.80	mmol/L
GeneXpert MTB/RIF	negative	-
Aspergillus serology	negative	-
Sputum culture	Positive >10^7^Pseudomonas aeruginosa	UFC/ml

Arterial blood gas analysis revealed a PaO₂ of 77.2 mmHg, a PCO₂ of 31 mmHg, a pH of 7.40, and an SpO₂ of 96% on room air, indicating satisfactory gas exchange.

The patient was treated with dual antibiotic therapy based on antibiogram results: imipenem 1 g every eight hours for seven days and amikacin 500 mg daily for five days, combined with chest physiotherapy for bronchial drainage, resulting in favorable clinical outcomes.

Upon discharge, preventive measures were prescribed, including seasonal vaccination, adequate hydration, continuous respiratory physiotherapy, and regular dental care. The patient has been followed up regularly every three months. She has remained clinically stable with adequate oxygen saturation, no signs of recurrent infection, and maintains regular physical activity. Follow-up imaging revealed unchanged radiologic findings, indicating stability of the pulmonary lesions.

Based on the clinical and radiological findings, the diagnosis of Kartagener syndrome was confirmed, with the exceptional complication of a destroyed lung. Her sister was then admitted for screening.

Case 2

I.S., a 26-year-old female, was born to a first-degree consanguineous marriage. Her sister was recently diagnosed with Kartagener syndrome, leading to her admission for screening. She was hospitalized at seven days of life for neonatal management of a maternal-fetal infection. She had a history of chronic nasal obstruction and anosmia.

The patient had been suffering from a chronic daytime and nocturnal cough since infancy, with expectoration during the daytime for one year.

On clinical examination, the patient was eupneic and SpO2 was 98%. Lung auscultation revealed bilateral rhonchi. Cardiac auscultation revealed heart sounds on the right side of the chest.

**Figure 4:**
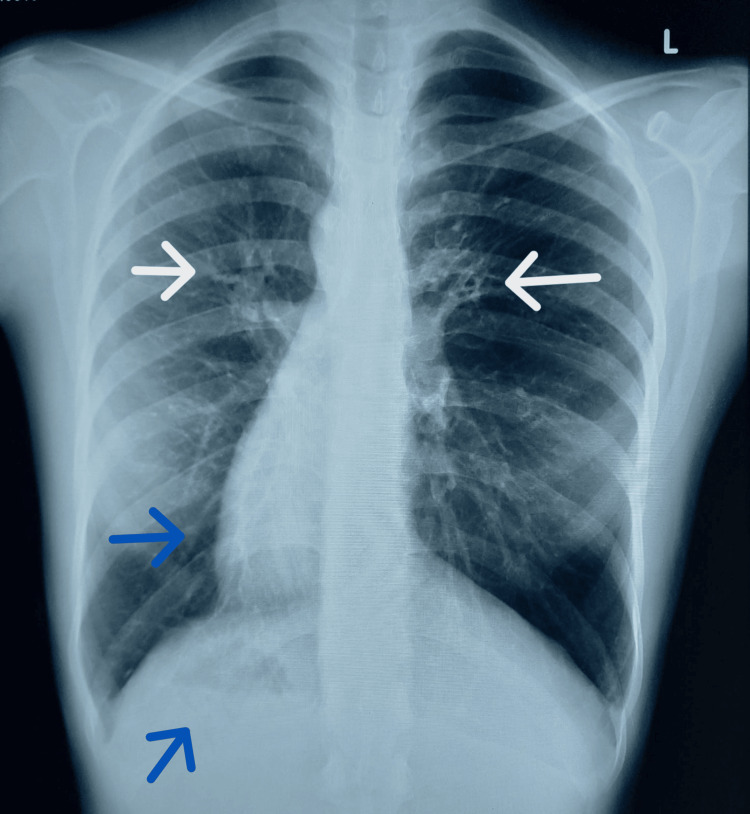
Chest X-ray The chest X-ray showed the cardiac silhouette, aortic knuckle, and gastric air bubble on the right side (blue arrow), suggesting situs inversus, with bilateral localized opacities and ring-like shadows (white arrow).

Chest X-ray showed the cardiac silhouette and stomach air on the right side, suggesting situs inversus, with bilateral localized opacities and ring-like shadows (Figure 4).

**Figure 5:**
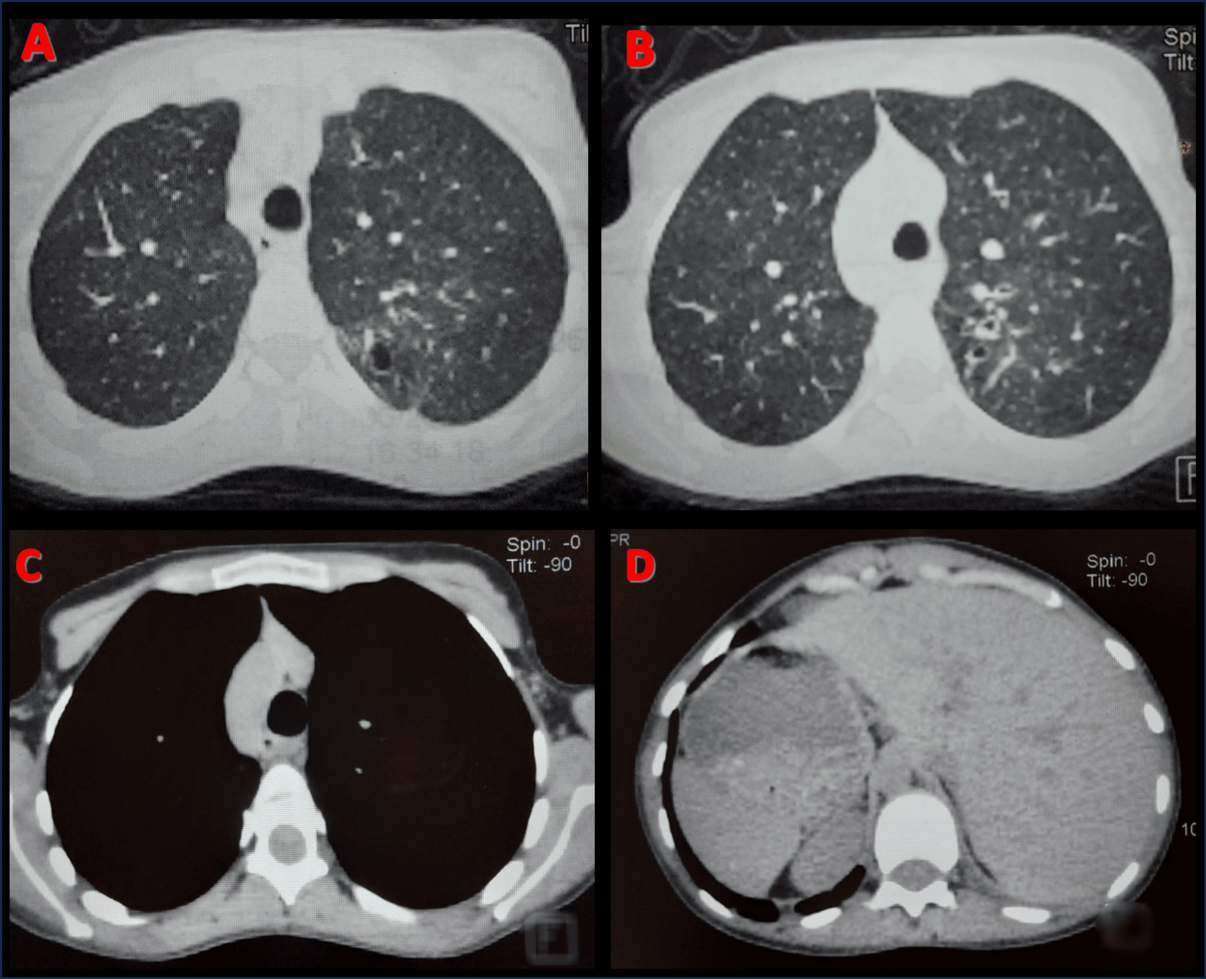
Thoracoabdominal CT scan Thoracoabdominal CT revealed cystic bronchiectasis (A and B), in the setting of complete situs inversus (trilobed left lung, bilobed right lung, transposed mediastinal vessels, right aortic arch, left-sided liver, and right-sided spleen) (C and D).

**Figure 6:**
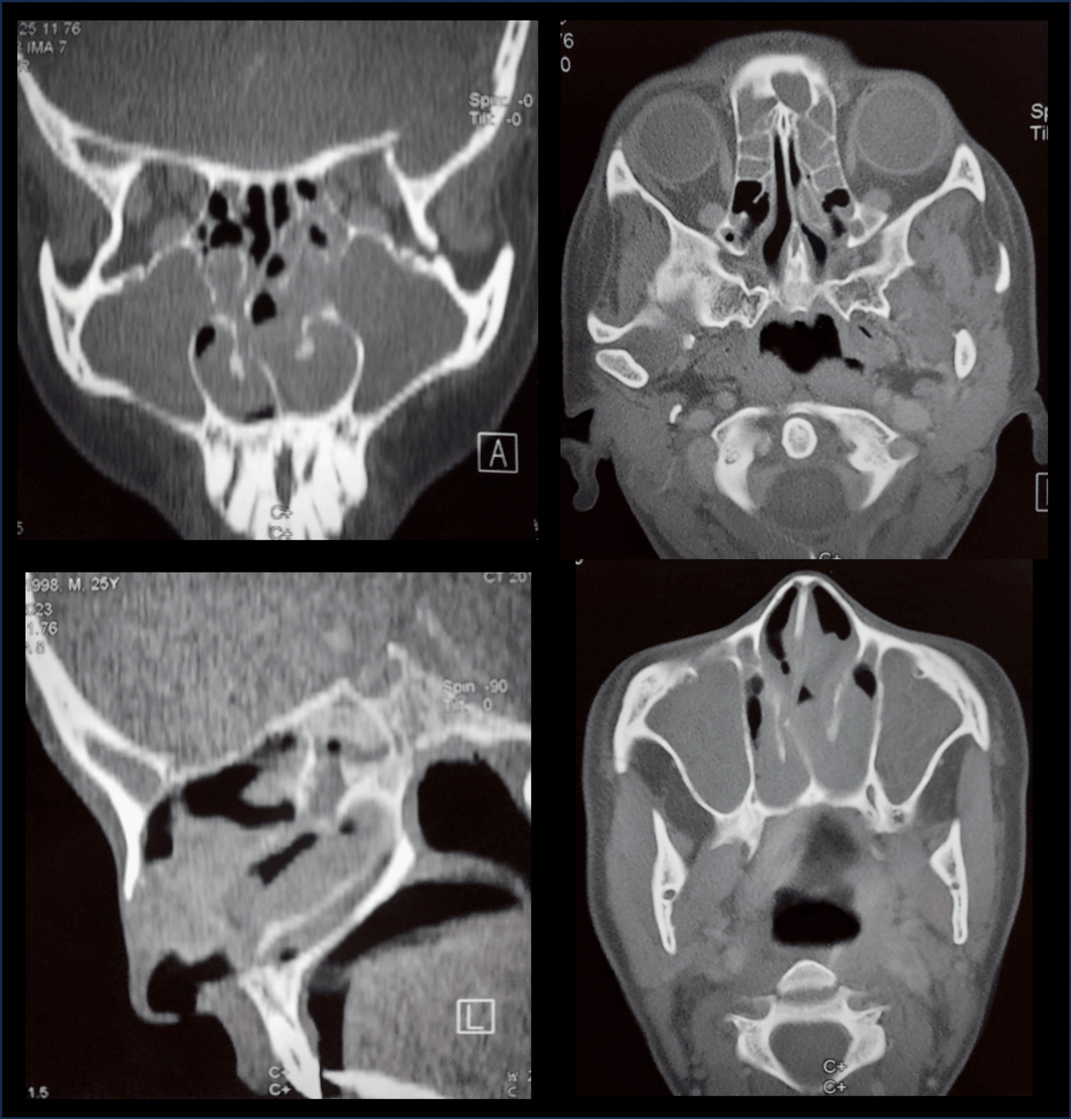
Sinus CT scan showed nasal polyposis.

Thoracoabdominal CT revealed cystic bronchiectasis with mucoid impactions, in the setting of complete situs inversus (trilobed left lung, bilobed right lung, transposed mediastinal vessels, right aortic arch, left-sided liver, and right-sided spleen) (Figure 5) and sinus CT showed nasal polyposis (Figure 6).

The clinico-radiological triad (situs inversus, bronchiectasis, and chronic sinusitis) confirms the diagnosis of Kartagener syndrome in the sister too.

## Discussion

KS, a subset of primary ciliary dyskinesia, is characterised by the triad of bronchiectasis, chronic sinusitis, and situs inversus [1]. Diagnosis is based on a combination of clinical features and, when available, confirmatory tests such as nasal nitric oxide measurement, ciliary ultrastructure analysis by electron microscopy, or genetic testing. However, in typical presentations, clinical diagnosis alone is sufficient. In our case, the diagnosis was made in accordance with the European Respiratory Society (ERS) guidelines, which support a clinical diagnosis in typical cases [2].

Delayed diagnosis of Kartagener syndrome is frequently reported in the literature, often due to non-specific symptoms during childhood or limited access to diagnostic tools. Several studies have shown that some patients are not diagnosed until adulthood [5]. Our case aligns with this pattern, with the diagnosis established at the ages of 20 and 26. Despite both sisters having a history of neonatal hospitalization for the management of a maternal-fetal infection, an etiological diagnosis was not established at that time. This is consistent with the study by Coren et al., which showed that the diagnosis was not established during the neonatal period despite the presence of neonatal respiratory distress in 37 out of 55 cases, situs inversus in 38 out of 55 cases, and early-onset troublesome rhinitis in 42 out of 55 cases. This underscores the frequent delay in diagnosis, even when typical symptoms are present early in life [6].

In KS, clinical presentation and disease severity can differ even within the same family, as documented in the literature [3,7]. This is consistent with our report, which highlights significant intra-familial phenotypic variability: while both sisters shared situs inversus, chronic rhinosinusitis and bronchiectasis, only the younger sibling progressed to a severely damaged, destroyed lung. This contrast between siblings shows the important role of screening, early diagnosis and long-term monitoring, especially in consanguineous families where genetic risk is higher [7]. Genetic testing can facilitate early familial screening by identifying pathogenic mutations in an index case, which allows for targeted testing of siblings and other at-risk family members, potentially leading to earlier diagnosis and timely intervention before the onset of irreversible organ damage [8,9]. However, in our case, genetic testing could not be performed due to financial constraints.

As is well known, Kartagener syndrome can progress to end-stage lung disease with irreversible chronic respiratory failure. However, it is important to distinguish between the terms 'destroyed lung syndrome' and 'end-stage lung disease.' Destroyed lung syndrome refers to a clinico-radiological condition with complete destruction and severe volume loss of one lung, often with compensatory hyperinflation of the contralateral lung, and not necessarily associated with respiratory failure. On the other hand, end-stage lung disease is a functional diagnosis characterized by severe chronic respiratory failure requiring long-term oxygen therapy and, in some cases, lung transplantation, even in the absence of extensive parenchymal destruction [10,11]. In our case, the younger sibling had a destroyed lung with normal oxygen saturation and preserved PaO₂, indicating that she has not yet reached the stage of end-stage lung disease. In the literature, severe involvement in Kartagener syndrome has only been described in the context of surgical interventions, particularly lobectomy for localized bronchiectasis [10] and lung transplantation in end-stage KS with end-stage respiratory failure [11]. However, the presence of a completely destroyed lung as a complication of KS has not been clearly documented to date.

The management of advanced forms may involve surgical intervention, particularly in cases of important clinical and functional deterioration [11,12]. In our case, although the patient had complete destruction of the right lung, the left lung exhibited compensatory hyperinflation, and preserving near-normal respiratory function. As a result, we chose a conservative approach focused on infection prevention including seasonal vaccination, adequate hydration, continuous respiratory physiotherapy, and regular dental care with close clinical follow-up. The patient's condition remained stable with clinical improvement and unchanged imaging findings with preserved PaO₂ during follow-up, supporting the decision to avoid immediate surgery in favor of continued conservative management in the absence of disease progression.

A multidisciplinary approach involving pulmonologists, ENT specialists, and respiratory therapists is essential, with appropriate management strategies such as airway clearance therapy, prompt antibiotic treatment, and regular imaging to help prevent disease progression and avoid irreversible pulmonary damage [13].

## Conclusions

This report describe a destroyed lung as a rare complication in Kartagener syndrome. It also highlights the phenotypic variability of the syndrome within the same family. Hence the importance of screening siblings, particularly in consanguineous families. Early management is crucial to reduce morbidity associated with recurrent infections and progressive lung damage.

## References

[1] Afzelius BA (1976). A human syndrome caused by immotile cilia. Science.

[2] Lucas JS, Barbato A, Collins SA (2017). European Respiratory Society guidelines for the diagnosis of primary ciliary dyskinesia. Eur Respir J.

[3] Sato M, Fujita Y, Imataka G, Kuwashima S, Takeuchi K, Yoshihara S (2024). Primary ciliary dyskinesia with identical genotype but distinct phenotypes in two siblings. Tohoku J Exp Med.

[4] Osarenkhoe J, Aiwuyo H, Aisosa O (2022). Destroyed lung syndrome: a review of 31 published cases. Open J Resp Dis.

[5] Tsetsou I, Balomenos V, Koreas P, Biliara IE, Tavernaraki K (2024). Late diagnosis of Kartagener syndrome in an adult female. Cureus.

[6] Coren ME, Meeks M, Morrison I, Buchdahl RM, Bush A (2002). Primary ciliary dyskinesia: age at diagnosis and symptom history. Acta Paediatr.

[7] Marafie MJ, Al Suliman IS, Redha AM, Alshati AM (2015). Primary ciliary dyskinesia: Kartagener syndrome in a family with a novel DNAH5 gene mutation and variable phenotypes. Egypt J Med Hum Genet.

[8] Knowles MR, Daniels LA, Davis SD, Zariwala MA, Leigh MW (2013). Primary ciliary dyskinesia. Recent advances in diagnostics, genetics, and characterization of clinical disease. Am J Respir Crit Care Med.

[9] Horani A, Ferkol TW (2018). Advances in the genetics of primary ciliary dyskinesia: clinical implications. Chest.

[10] Lin H, Cao Z, Zhao X, Ye Q (2016). Left middle lobectomy for bronchiectasis in a patient with Kartagener syndrome: a case report. J Cardiothorac Surg.

[11] Wang B, Zhang X, Jiang W (2020). Double lung transplantation for end-stage Kartagener syndrome: a case report and literature review. J Thorac Dis.

[12] Marro M, Leiva-Juárez MM, D'Ovidio F (2023). Lung transplantation for primary ciliary dyskinesia and Kartagener syndrome: a multicenter study. Transpl Int.

[13] Shapiro AJ, Zariwala MA, Ferkol T (2016). Diagnosis, monitoring, and treatment of primary ciliary dyskinesia: PCD foundation consensus recommendations based on state of the art review. Pediatr Pulmonol.

